# Subclinical Doses of Combined Fumonisins and Deoxynivalenol Predispose *Clostridium perfringens–*Inoculated Broilers to Necrotic Enteritis

**DOI:** 10.3389/fphys.2022.934660

**Published:** 2022-07-22

**Authors:** R. Shanmugasundaram, D. Adams, S. Ramirez, G. R. Murugesan, T. J. Applegate, S. Cunningham, A. Pokoo-Aikins, A. E. Glenn

**Affiliations:** ^1^ Toxicology and Mycotoxin Research Unit, U.S. National Poultry Research Center, Agricultural Research Service, U.S. Department of Agriculture, Athens, GA, United States; ^2^ Department of Poultry Science, University of Georgia, Athens, GA, United States; ^3^ DSM Animal Nutrition and Health, Kaiseraugst, Switzerland

**Keywords:** fumonisins, deoxynivalenol, necrotic enteritis, tight junction proteins, immune response, broiler chicken

## Abstract

Fumonisins (FB) and deoxynivalenol (DON) are mycotoxins which may predispose broiler chickens to necrotic enteritis (NE). The objective of this study was to identify the effects of subclinical doses of combined FB and DON on NE. A total of 480 day-old male broiler chicks were divided into four treatment groups; 1) control group (basal diet + *Clostridium perfringens*); 2) necrotic enteritis group (basal diet + *Eimeria maxima + C. perfringens*); 3) FB + DON group (basal diet + 3 mg/kg FB + 4 mg/kg DON + *C. perfringens*); and 4) FB + DON + NE group (basal diet + 3 mg/kg FB + 4 mg/kg DON + *E. maxima + C. perfringens*). Birds in NE and FB + DON + NE groups received 2.5 × 10^3^ *E. maxima* on day 14. All birds were inoculated with *C. perfringens* on days 19, 20, and 21. On day 35, birds in the NE, FB + DON, and FB + DON + NE groups had 242, 84, and 339 g lower BWG and a 19-, 2-, and 22-point increase in FCR respectively, than in the control group. Subclinical doses of FB + DON increased (*p* < 0.05) the NE lesion scores compared to the control group on day 21. On day 21, birds in the NE, FB + DON, and FB + DON + NE groups had increased (*p* < 0.05) serum FITC-D, lower (*p* < 0.05) jejunal tight junction protein mRNA, and increased (*p* < 0.05) cecal tonsil IL-1 mRNA compared to control group. On day 21, birds in the NE group had decreased (*p* < 0.05) villi height to crypt depth ratio compared to the control group and the presence of FB + DON in NE-induced birds further decreased the villi height to crypt depth ratio. Birds in the NE, FB + DON, and FB + DON + NE groups had increased (*p* < 0.05) *C. perfringens*, lower (*p* < 0.05) *Lactobacillus* loads in the cecal content, and a lower (*p* < 0.05) CD8^+^: CD4^+^ cell ratio in the cecal tonsils compared to the control group. It can be concluded that subclinical doses of combined FB and DON predispose *C. perfringens-*inoculated birds to NE, and the presence of FB + DON in NE-induced birds exacerbated the severity of NE.

## Introduction

Corn is one of the major components of poultry feed, and up to 65% of finished poultry feed can be comprised of corn and corn byproducts (Alqaisi et al., 2017). Poultry diets are often contaminated with more than one mycotoxin. Fumonisins (FB) and deoxynivalenol (DON) are secondary mycotoxin metabolites produced by *Fusarium verticillioides* and *Fusarium graminearum*, respectively (Glenn, 2007). According to the 2021 survey by Biomin, FB and DON are the most prevalent mycotoxins in poultry feed samples in North America and were detected in 64% and 47% of poultry diets, respectively (Biomin, 2021). Recent surveys have identified that, on average, the amount of DON in corn and cereal grain was 808 μg/kg and 1,721 μg/kg, respectively; the amount of FB in corn was 2,405 μg/kg. Furthermore, DON and FB can co-occur in poultry feed ingredients, and 92% of feed samples analyzed in 2021 had more than one mycotoxin (Biomin, 2021). Though negative effects of FB have been reported when FB are present at 100 mg/kg in chicken feed, FB has been suggested to cause negative effects even at a lower dose when co-occurring with other mycotoxins such as aflatoxins, DON, and zearalenone in poultry (Ogbuewu, 2011). Co-occurrence of mycotoxins decreases the tolerance to individual mycotoxins and, therefore, the existence of multiple mycotoxins in poultry feed even at subclinical levels can be expected to exacerbate the pathology of individual mycotoxins in poultry.

European Food Safety Authority (EFSA) and Food and Drug Administration (FDA) have set guidelines for maximal permissible levels of major mycotoxins in poultry feed. However, subclinical doses of FB (20 mg/kg diet) and DON (5 mg/kg diet), alone (Antonissen et al., 2014; Antonissen et al., 2015) or in combination (Grenier et al., 2016), cause metabolic and immunological disturbances that amplify the severity of necrotic enteritis (NE), coccidiosis, and increase the susceptibility to bacterial diseases in chickens. Mycotoxin interactions within the animal system are mainly additive, but depending on the endpoint assessment these interactions can also be synergistic or antagonistic (Grenier and Oswald, 2011).

Currently, NE is an economically important disease affecting the modern broiler industry. Subclinical NE affects broilers between 2–5-weeks of age and is characterized by intestinal mucosal damage, with no apparent clinical signs or mortality (Hofacre et al., 2018). Subclinical NE leads to decreased digestion and absorption of nutrients, reduced weight gain, and impaired feed conversion rate in poultry (Immerseel et al., 2004). Coccidiosis and feed contaminated with mycotoxins, particularly FB and DON (Antonissen et al., 2015), are considered to be the predisposing factors for NE. In addition, mycotoxins reduce the efficacy of coccidiosis vaccines and, therefore, contribute to NE incidence in chickens (Broom, 2017). Recent restrictions on the use of antibiotics and ionophores in broiler production led to an increase in the occurrence of NE by altering the composition and microbial balance in the gut microbiome (Smith, 2019). The causative organism for NE is *Clostridium perfringens,* a commensal bacterium in the gastrointestinal tract of healthy broilers. *C*. *perfringens* loads range up to 1 × 10^5^ CFU/g of digesta in healthy chickens, while in chickens with clinical NE symptoms, *C. perfringens* loads increase to 1 × 10^6^ to 1 × 10^8^ CFU/g of digesta, along with associated toxins that include necrotic toxin enteritis B-like (NetB) (Timbermont et al., 2011; Mora et al., 2020).

In the past, FB below 50 mg/kg feed and DON at 5 mg/kg feed were considered not to cause negative effects in poultry (Dänicke et al., 2001; Filazi et al., 2017). However, recent studies have identified that a combined dose of 20 mg/kg FB and 1.5 mg/kg DON decreases the production performances, causes gut damage, and increases coccidiosis severity (Antonissen et al., 2014; Antonissen et al., 2015; Grenier et al., 2016), which can be expected to predispose the broilers to NE. Information regarding the role of chronic exposure of subclinical doses, even at doses much lower than previous studies, of mycotoxins is lacking. Continuous exposure to mycotoxins is expected to damage the gut wall and increase gut permeability to negatively affect the FDA recommendation on NE, gut health, and immune response in chickens. Therefore, the objective of this study was to evaluate the combined effects of FB (3 mg/kg diet) and DON (4 mg/kg diet) on gut health and immune parameters and evaluate the role of mycotoxins as a predisposing factor in inducing and increasing the severity of a NE in poultry.

## Materials and Methods

### Diet Formulation

A non-medicated corn–soybean meal-based mash diet was applied as a basal diet (Table 1). The feeding study was divided into two experimental phases: 1) d0–18, starter feed, and 2) d19–35, finisher feed. Two strains of *Fusarium*, *F. graminearum* strain PH-1 and *F. verticillioides* strain M3125 were cultured for DON and FB production, respectively (Altpeter and Posselt, 1994). In brief, *Fusarium* strains were cultured separately in carboxymethyl cellulose liquid media and shaken for 5 days (*F. verticillioides*) or 7 days (*F. graminearum*), and spores were collected. Fungal spores were added separately to rice media and incubated until mycotoxin content was analyzed. The homogenized rice cultures with FB and DON were mixed with a small portion of the basal diet and re-mixed with the appropriate amount of basal feed to create the experimental diets. The starter diet (d0–18) and the finisher diet were formulated to contain 3 mg/kg FB and 4 mg/kg DON, respectively. The final diets were analyzed by LC-MS-MS to determine the actual content of FB and DON and the content of other major mycotoxins (Romer Labs, Union, MO, United States). The mycotoxin content of the formulated experimental diet is provided in Table 2.

**TABLE 1:** Ingredient and nutrient composition of basal diets (as-fed basis).

Ingredients (%)	Starter	Finisher
Corn	56.29	64.86
Soybean meal, 48% CP	37.87	28.44
Soybean oil	2.18	3.80
Dicalcium phosphate	1.48	0.84
Calcium carbonate	0.91	0.78
Sodium chloride	0.40	0.40
MHA	0.37	0.32
L-lysine	0.21	0.22
Trace mineral premix	0.10	0.10
Choline chloride (60%)	0.07	0.08
L-threonine	0.06	0.07
Vitamin premix	0.05	0.05
Phytase (500 ftu)	0.01	0.01

Nutrients, vitamins, and minerals were provided in the form and amount described in the NRC, standard reference diet for chickens (Council, 1994).

**TABLE 2:** Analyzed mycotoxin content of experimental diets.

	Aflatoxin (ppm)	Fumonisin (ppm)	Deoxynivalenol (ppm)	Zearalenone (ppm)	Nivalenol (ppm)
Starter diet					
Control	0.04^a^	0.4^b^	0.1	<0.05	<0.1
Treatment	0.03^a^	2.8^b^	4.3	0.3	<0.1
Finisher diet					
Control	0.003^a^	1.5^b^	0.2	0.07	<0.1
Treatment	0.002^a^	2.9^b^	4.0	0.4	0.2

a Total aflatoxins (B1 + B2).

b Total fumonisins (B1 + B2 + B3).

The final diets were analyzed by LC-MS/MS, at Romer Labs, Union, MO, United States.

### Birds and Housing

This 35-day feeding trial was conducted with 480 day-old male Ross × Ross 708 strain broiler chicks (Aviagen, Blairsville, GA, United States). The animal care practices and use procedures were followed under the Guide for the Care and Use of Agricultural Animals in Research and Teaching (McGlone, 2010). All animal protocols were approved by the Institutional Animal Care and Use Committee at the Southern Poultry Research Group, Athens, GA. The birds were raised under the supervision of a licensed poultry veterinarian. All birds were euthanized by methods approved by the American Veterinary Medical Association (AVMA). Day-old broiler chicks were raised in 1.5 m × 1.5 m floor pens (stocking density of 15 birds/m^2^) on new shavings/litter following standard industry practice in North America and raised under ambient humidity. Chickens were weighed individually and randomly distributed into either one of the four treatment groups. The experimental treatment groups were 1) control group (basal diet + *C. perfringens* challenge), 2) NE group (basal diet + *E. maxima + C. perfringens*), 3) FB + DON group (basal diet + 3 mg/kg FB +4 mg/kg DON + *C. perfringens*), and 4) FB + DON + NE group (basal diet + 3 mg/kg FB + 4 mg/kg DON + *E. maxima + C. perfringens*). Each treatment was replicated in 8 pens with 15 birds/pen in a completely randomized design. Chicks had *ad libitum* access to the feed and water throughout the experimental period. The mortality of the birds was recorded daily. The birds were housed in floor pens equipped with nipple-type waterers and thermostatically controlled heaters.

### Production Performances and NE Lesion Score

On day 14, 2.5 × 10^3^ *Eimeria maxima* sporulated oocysts/bird were mixed in the feed of NE and FB + DON + NE groups. On days 19, 20, and 21, birds in all treatment groups were challenged with 1 × 10^8^ CFU/bird *C. perfringens* (strain #6) through the feed to target 3%–5% NE mortality as described earlier (Hofacre et al., 1998). Before the *C. perfringens* challenge, feed and water were withdrawn for 4 h and 2 h, respectively. Three birds from each pen were randomly sacrificed and examined for the NE lesion score on day 21. Lesion scoring was based on a 0 to 3 scale as described earlier (Hofacre et al., 1998), wherein 0 is normal, 1 is a slight mucus covering the small intestine, 2 is a necrotic small intestinal mucosa, and 3 is a sloughed cells and blood in the small intestinal mucosa and contents. Bodyweight and feed intake were measured at 0, 7, 14, 21, 27, and 35 days of age. Average feed intake and body weight gain (BWG) were corrected for mortality for calculating the feed conversion ratio (FCR) for each pen.

### Gut Permeability to FITC-Dextran

Gut permeability was measured using the FITC-D assay as described earlier (Kuttappan et al., 2015). On days 21, 28, and 35, one bird/pen (*n* = 8) was orally gavaged with 1 ml of fluorescein isothiocyanate dextran (FITC-D, MW 4000; Sigma-Aldrich, United States) 2.2 mg/bird. 2 h later, the birds were euthanized, and blood was collected by cardiac puncture. Blood samples were centrifuged at 450 × g for 10 min to separate the serum from red blood cells. The serum was diluted in PBS with pH 7.4 at a 1:1 ratio. The serum FITC-D concentration was determined based on a standard curve. A standard curve with 0, 0.2, 0.4, 0.6, 0.8, 1.0, and 2 μg/ml FITC-D was drawn using Gen5 software on the same plate as the samples. The samples and standards were measured at an excitation wavelength of 485 nm and emission wavelength of 528 nm (Synergy HT, multi-mode microplate reader, BioTek Instruments, Inc., VT).

### Spleen and Cecal Tonsil CD8^+^: CD4^+^ Ratio

On days 21, 28, and 35, post-challenge, the effect of FB and DON on the spleen and cecal tonsil CD4^+^ and CD8^+^ cell percentages were determined by flow cytometry as described previously (Shanmugasundaram et al., 2015). In brief, single-cell suspensions from the spleen and cecal tonsils were enriched for mononuclear cells by density centrifugation over Histopaque (1.077 g/ml, Sigma-Aldrich, St. Louis, MO) for 15 min at 400 g. The cells were incubated with a 1:250 dilution of fluorescent-isothiocyanate conjugated mouse anti-chicken CD4^+^ (Southern Biotech, Birmingham, AL), 1:450 dilution of phycoerythrin-conjugated mouse anti-chicken CD8^+^ (Southern Biotech, Birmingham, AL), and 1:200 dilution of unlabeled mouse IgG for 15 min. The unbound antibodies were removed by centrifugation, the percentages of CD4^+^ and CD8^+^ cells were analyzed using a flow cytometer (Guava EasyCyte, Millipore, MA), and CD8^+^: CD4^+^ ratio was calculated.

### Jejunal Tight Junction Protein and Cecal Tonsil Cytokine mRNA Expression

On days 21, 28, and 35, 1 bird per pen (*n* = 8) was euthanized by cervical dislocation. A portion of distal-jejunum and proximal ileum (1 cm proximal and 1 cm distal to the Meckel’s diverticulum) and cecal tonsils were collected in cryovials containing RNAlater^®^ (Ambion Inc., Austin, TX, United States) and stored at −70°C until further analysis. The jejunum was analyzed for claudin-1, claudin-2, and zona-occluden-1 tight junction protein mRNAs, and cecal tonsils were analyzed for pro-and anti-inflammatory cytokines IL-1β, IL-10, LITAF, and IFN-γ mRNA expression, as described previously (Shanmugasundaram and Selvaraj, 2012).

Total RNA was extracted from all experimental groups using the TRI reagent (Molecular Research Center, Cincinnati, OH) following the manufacturer’s instructions. RNA concentration and purity were determined using an Epoch spectrophotometer (BioTek, Winooski, VT, United States), using the 260/280 and 260/230 ratios. 2 mg RNA was reverse transcribed into cDNA and analyzed for IL-1β, IL-10, LITAF, IFN-γ, claudin-1, claudin-2, and zona-occluden-1 by real-time PCR (CFX96 Touch Real-Time System, BioRad, Hercules, CA) using SYBR Green. Primer sequences and annealing temperature are provided in Table 3. Each well contained 10 µl SYBR Green PCR master mix, 7 µl RNAse-free water using C1000 TouchTM Thermal cycler (BioRad, Hercules, CA), 2 µl (∼600 ng/μl) cDNA, 0.5 µl forward primer (5 µM), and 0.5 µl reverse primer (5 µM). To perform real-time PCR, the following settings were used for all genes: an initial denaturation of 95°C for 10 min (1 cycle); followed by 95°C for 15 s; and 60°C for 45 s (40 cycles). The melting profile was determined by heating samples at 65°C for 30 s and then increasing the temperature at a linear rate of 10°C/s to 95°C while continuously monitoring fluorescence. Housekeeping genes of β-actin, glyceraldehyde-3-phosphate dehydrogenase (GAPDH), and ribosomal protein S13 (RPS13) were selected, and the stability was analyzed using Normfinder software (Department of Molecular Medicine, Aarhus University Hospital, Denmark) as described previously (Shanmugasundaram et al., 2018). The RPS13 gene was selected for data normalization because it was the most stable expression among the set of housekeeping genes analyzed for normalization. The cecal tonsil IL-1β, IL-10, LITAF, and IFN-γ, the jejunal claudin-1, claudin-4, and zona-occluden-1 mRNAs were normalized with RPS13. The 2ˉ^∆∆Ct^ method, as previously described (Livak and Schmittgen, 2001), where Ct is the threshold cycle, was used to calculate the mRNA fold change. The fold change was calculated as 2^(Ct Sample – housekeeping)^/2 ^(Ct Reference – housekeeping)^. The reference group was the control group.

**TABLE 3:** Primers and PCR conditions for PCR.

Gene	Primer sequence1 (5′- 3′)	Annealing temperature (°C)	Reference
IL1-β	F: TCC​TCC​AGC​CAG​AAA​GTG​A	57.5	Shanmugasundaram and Selvaraj. (2012)
	R: CAG​GCG​GTA​GAA​GAT​GAA​GC		
IL10	F: CATGCTGCTGGGCCTGAA	57.5	Shanmugasundaram and Selvaraj. (2012)
	R: CGT​CTC​CTT​GAT​CTG​CTT​GAT​G		
LITAF	F: ATC​CTC​ACC​CCT​ACC​CTG​TC	55	Markazi et al. (2019)
	R: GGC​GGT​CAT​AGA​ACA​GCA​CT		
IFN-γ	F: GGC​GTG​AAG​AAG​GTG​AAA​GA	57.4	Shanmugasundaram et al. (2021)
	R: CCT​CTG​AGA​CTG​GCT​CCT​TTT		
RPS-13	F: CAA​GAA​GGC​TGT​TGC​TGT​TCG	55	Shanmugasundaram et al. (2019c)
	R: GGC​AGA​AGC​TGT​CGA​TGA​TT		
Claudin-1	F: CAT​ACT​CCT​GGG​TCT​GGT​TGG​T	55	Chen et al. (2017)
	R: GAC​AGC​CAT​CCG​CAT​CTT​CT		
Claudin-2	F: CCT​GCT​CAC​CCT​CAT​TGG​AG	55	Li et al. (2015)
	R: GCT​GAA​CTC​ACT​CTT​GGG​CT		
Zona occluden-1	F: TGT​AGC​CAC​AGC​AAG​AGG​TG	56	Zhang et al. (2017)
	R: CTG​GAA​TGG​CTC​CTT​GTG​GT		
*C. perfringens*	F: AAA​GGA​AGA​TTA​ATA​CCG​CAT​AA	55	Shanmugasundaram et al. (2020)
	R: ATC​TTG​CGA​CCG​TAC​TCC​CC		
*Lactobacillus*	F: CAT​CCA​GTG​CAA​ACC​TAA​GAG	55	Wang et al. (1996)
	R: CCACCGTTACACCGGGAA		
*Bifidobacterium*	F: GGGTGGTAATGCCGGATG	57	Langendijk et al. (1995)
	R: CCACCGTTACACCGGGAA		

### *C. perfringens*, Total *Lactobacillus,* and Total *Bifidobacteria* Loads in the Cecal Content

On days 21, 28, and 35, cecal content from one bird/pen (*n* = 8) was collected and stored at −20°C until further use. The DNA from the cecal microflora DNA was extracted as described earlier (Amit-Romach et al., 2004; Shanmugasundaram et al., 2019a). The DNA pellet was resuspended in TE buffer (10 mM Tris-HCl, 1 mM EDTA, pH 8.0) and stored at −20°C until further use. The final concentration of the isolated DNA was determined using an Epoch spectrophotometer (BioTek, Winooski, VT, United States). The DNA samples were diluted to a final concentration of 100 ng/μl. The primers for *Lactobacillus*, *Bifidobacterium*, and *C. Perfringens* were adapted from an earlier publication (Amit-Romach et al., 2004). The Ct values were converted into CFU/g using a standard curve as described previously (Shanmugasundaram et al., 2019b). The PCR efficiency and the slope and intercept of the standard curve were determined by the CFX software (Bio-Rad, Hercules, CA). The PCR efficiency of the *C. perfringens*, *Lactobacillus*, and *Bifidobacteria* standard curve analysis was 98%, 99%, and 99%, respectively.

### Jejunal and Ileal Histomorphology

On 21, 28, and 35 days, jejunal and ileal samples were collected from one bird/pen (*n* = 8) from each replication post-challenge. Approximately 4 cm of jejunal and ileal samples were cut proximal and distal to the Meckel’s diverticulum and stored in buffered formalin. The jejunal and ileal samples were processed at room temperature in a graded series of alcohols (15 min in 50% ethanol, 15 min in 70% ethanol, 15 min in 96% ethanol, and 30 min in 100% ethanol with one change at 15 min), cleared in Pro-par (Anatech, Battle Creek, MI) for 45 min with 2 changes at 15 and 30 min, and infiltrated with paraffin at 60°C overnight with one change at 15 min using a tissue processor (Sakura Finetek USA, Inc., Torrance, CA, United States). Paraffin blocks were cut into 5-μm cross-sections and mounted on super frost slides (Thermo Fisher Scientific, Waltham, MA, United States). Slides were then stained with hematoxylin and eosin. Cross-sections were viewed using the cellSens Imaging software (Olympus America, Central Valley, PA) to measure villi length and crypt depth. Ten intact lamina propria villi and crypts per section and 5 sections per sample were analyzed as described earlier (Shanmugasundaram et al., 2020). The tip of the villus to the villus–crypt junction was measured as villus height. The crypt depth was defined by the depth of the invagination between adjacent villi. All the samples in a time point were collected from the same bird, except for the gut permeability analysis for which a second bird was used.

### Statistical Analysis

A one-way ANOVA (JMP Pro 15 software, Cary, NC) was used to examine the effects of the subclinical dose of FB + DON on dependent variables, with the pen being considered as the experimental unit. When the main effects were significant (*p* < 0.05), differences between means were analyzed by Tukey’s least-square means comparison. Values reported are least-squares means ± SEM. The lesion scores were analyzed by a non-parametric test, and a Wilcoxon/Kruskal–Wallis rank-sum test was used to separate the means. The heatmap was rendered with JMP’s plotting library (Šefcová et al., 2020).

## Results

### Effect of Subclinical Dose of FB + DON and *E. maxima/C. perfringens* Challenge on Production Performances

**FIGURE 1:**
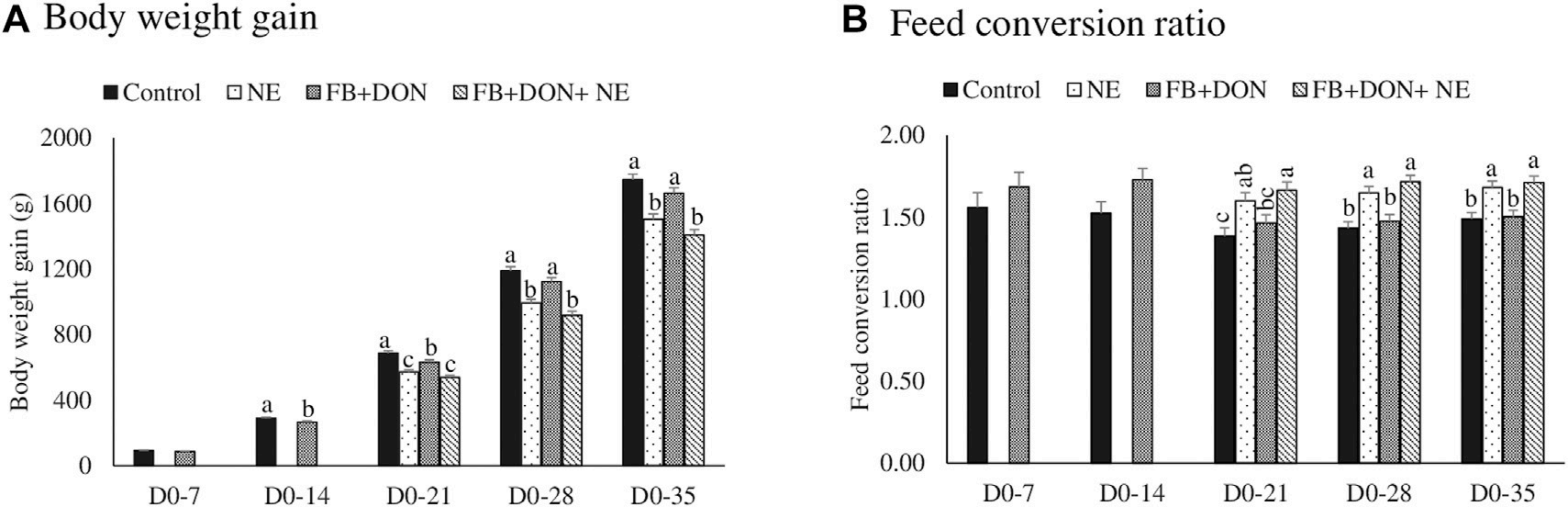
Effect of subclinical dose of FB + DON and *E. maxima/C. perfringens* challenge on production performances. Day-old chicks were distributed into four treatment groups: control, necrotic enteritis (NE), fumonisin + deoxynivalenol (FB + DON), and FB + DON + NE groups. Birds in the NE and FB + DON + NE groups received 2.5 × 10^3^ *Eimeria maxima* oocyst per bird on day 14. All birds received 1 × 10^8^ CFU/bird of *Clostridium perfringens* on days 19, 20, and 21. Body weight and feed consumption was measured on days 0, 7, 14, 21, 28, and 35 of age to calculate body weight gain (Panel 1A) and feed consumption ratio (Panel 1B). Mortality-corrected body weight gain and feed conversion ratio are presented. Bars (+SEM) without a common superscript differ significantly (*p* < 0.05). *n* = 8 pens of 15 birds/pen.

There were significant (*p* < 0.05) treatment effects on body weight gain on days 14, 21, 28, and 35 (Figure 1A). On day 14, birds in the FB + DON had lower BWG compared to the birds in the control group. On day 35, birds in the NE and FB + DON + NE groups had 242 g (*p* < 0.05) and 339 g (*p* < 0.05) lower BWG than the birds in the control group, respectively.

There were significant (*p* < 0.05) treatment effects on the FCR on days 21, 28, and 35 (Figure 1B). On day 14, birds in the FB + DON group had 21 points (*p* = 0.05) increase in FCR compared to the birds in the control group. On day 35, birds in the NE and FB + DON + NE groups had 19 points and 22 points significant increase in FCR than those in the control group.

### Effect of Subclinical Dose of FB + DON and *E. maxima/C. perfringens* Challenge on NE Lesion Score

There were significant (*p* < 0.05) treatment effects on the NE lesion score on day 21 (Table 4). Birds in the control group had the lowest Wilcoxon/Kruskal–Wallis score means for lesion scores. In birds with induced NE, 4.2% (1 out of 24) had a NE lesion score of 0, 58.3% (14 out of 24) had a NE lesion score of 1, 37.5% (9 out of 24) had a NE lesion score of 2, and 0% had a NE lesion score of 3. In birds exposed to FB + DON and induced with NE, 0% had a NE lesion score of 0 , 37.5% (9 out of 24) had a NE lesion score of 1, 62.5% (15 out of 24) had a NE lesion score of 2, and 0% had a NE lesion score of 3. Birds in the NE group had higher (*p* < 0.05) Wilcoxon/Kruskal–Wallis Score Means for lesion scores than scores observed in the control group. Subclinical dose of FB + DON increased (*p* < 0.05) the Wilcoxon/Kruskal–Wallis Score Means for lesion scores compared with the control group on day 21. The presence of FB + DON in NE-challenged birds increased (*p* < 0.05) the Wilcoxon/Kruskal–Wallis Score Means for lesion scores compared to the NE group.

**TABLE 4:** Effect of subclinical dose of FB + DON and *E. maxima/C. perfringens* challenge on necrotic enteritis lesion score at 21 days of age.

Treatment	Score 0	Score 1	Score 2	Score 3	Rank scores mean	Chi sq. *p*-value
Control	21	3	0	0	22.4	0.01
NE	1	14	9	0	60.5	
FB + DON	18	6	0	0	37.3	
FB + DON + NE	0	9	15	0	73.8	

Day-old chicks were distributed into four treatment groups: control, necrotic enteritis (NE), fumonisin + deoxynivalenol (FB + DON), and FB + DON + NE groups. Birds in the NE and FB + DON + NE groups received 2.5 × 10^3^ *Eimeria maxima* oocyst per bird on day 14. All birds received 1 × 10^8^ CFU/bird of *Clostridium perfringens* on days 19, 20, and 21. On day 21, three birds were scored for NE lesion scores on a 0 to 3 scale wherein 0 is normal, 1 shows slight mucus covering the small intestine, 2 has a necrotic small intestinal mucosa, and 3 shows sloughed cells and blood in the small intestinal mucosa and contents. Lesion scores were analyzed by a non-parametric test, and Wilcoxon/Kruskal–Wallis rank-sum test was used to separate the means.

### Effect of Subclinical Dose of FB + DON and *E. maxima/C. perfringens* Challenge on Gut Permeability to FITC-Dextran

**FIGURE 2:**
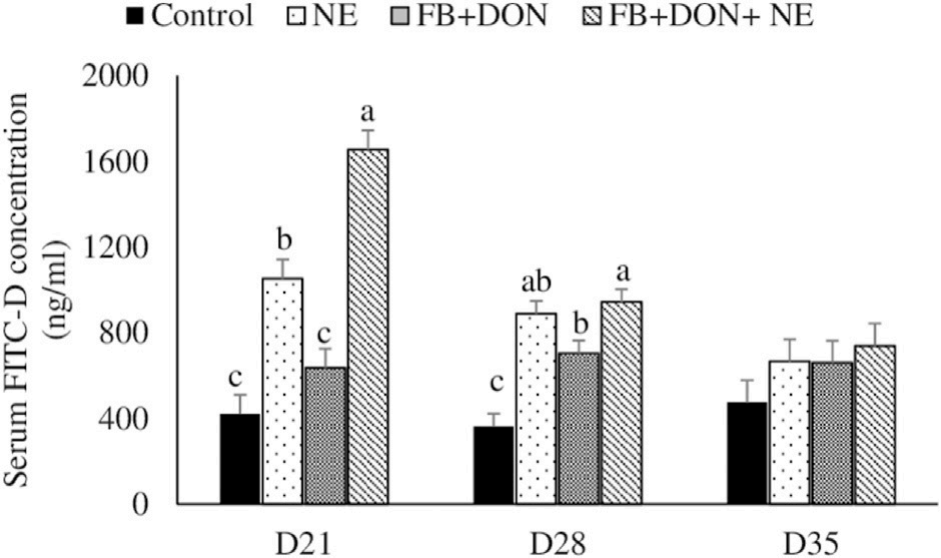
Effect of subclinical dose of FB + DON and *E. maxima/C. perfringens* challenge on gut permeability to FITC-dextran. Day-old chicks were distributed into four treatment groups: control, necrotic enteritis (NE), fumonisin + deoxynivalenol (FB + DON), and FB + DON + NE groups. Birds in the NE and FB + DON + NE groups received 2.5 × 10^3^ *Eimeria maxima* oocyst per bird on day 14. All birds received 1 × 10^8^ CFU/bird of *Clostridium perfringens* on days 19, 20, and 21. One bird/pen was orally gavaged with 2.2 mg/bird of 4,000 MW fluorescein isothiocyanate dextran (FITC-D), and blood was collected 2 h later. Serum FITC-D concentration was determined in a microplate reader. Bars (+SEM) without a common superscript differ significantly (*p* < 0.05). *n* = 8 (8 pens of 15 birds/pen).

There were significant (*p* < 0.05) treatment effects on the serum FITC-D concentration on days 21 and 28 (Figure 2). On day 21, birds in the NE, FB + DON, and FB + DON + NE groups had a 150% (*p* < 0.05), 51% (*p* > 0.05), and 293% (*p* < 0.05) increase in serum FITC-D compared to the birds in the control group. Similar trends were observed on day 28. The presence of FB + DON in NE-challenged birds increased (*p* < 0.05) the serum FITC-D concentration further by 57%, compared with NE group on day 21.

### Effect of Subclinical Dose of FB + DON and *E. maxima/C. perfringens* Challenge on Jejunal Tight Junction Protein mRNA Expression

**FIGURE 3:**
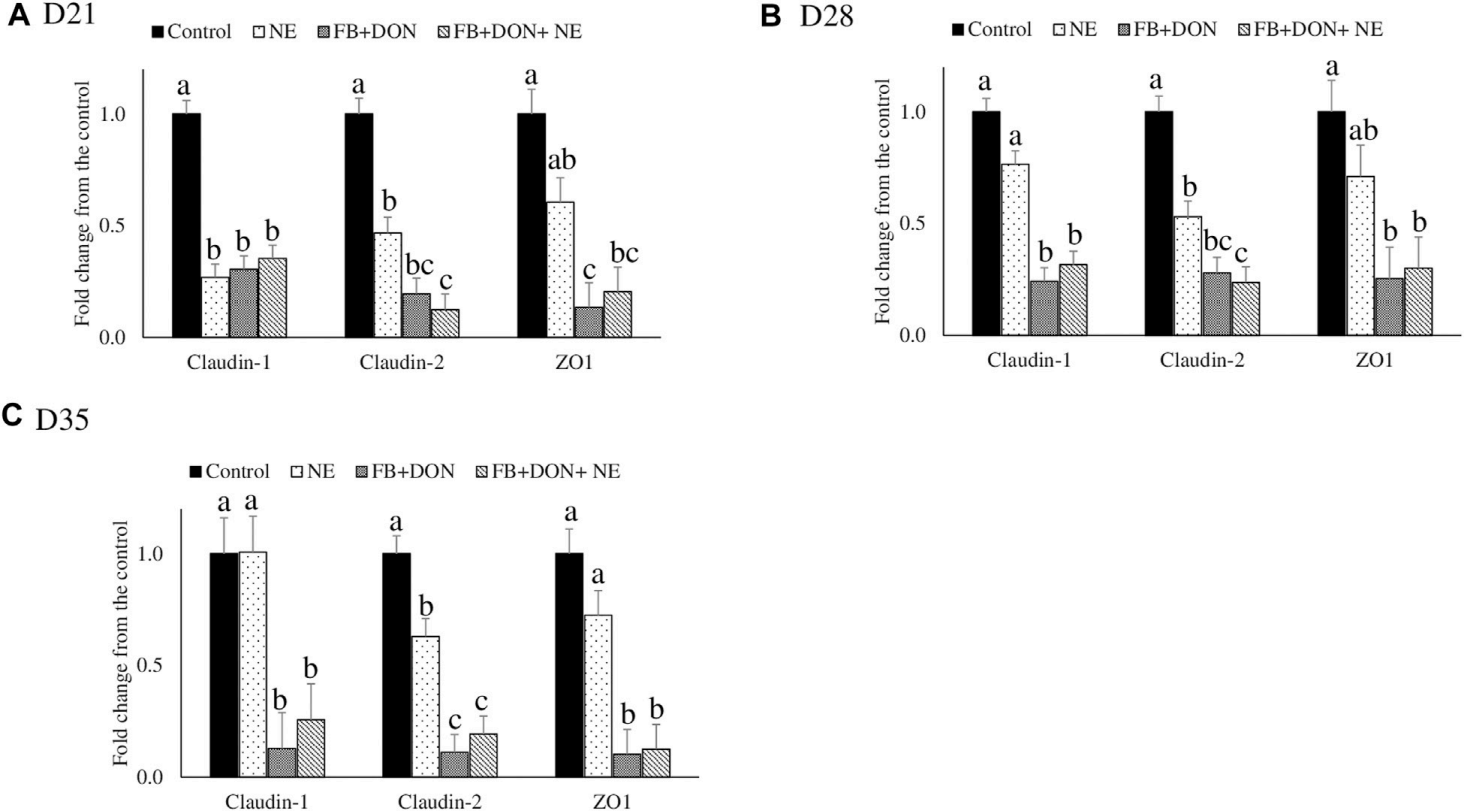
Effect of subclinical dose of FB + DON and *E. maxima/C. perfringens* challenge on jejunal tight junction protein mRNA expression. Day-old chicks were distributed into four treatment groups: control, necrotic enteritis (NE), fumonisin + deoxynivalenol (FB + DON), and FB + DON + NE groups. Birds in the NE and FB + DON + NE groups received 2.5 × 10^3^ *Eimeria maxima* oocyst per bird on day 14. All birds received 1 × 10^8^ CFU/bird of *Clostridium perfringens* on days 19, 20, and 21. Tight junction protein mRNA content was analyzed after correcting for the housekeeping gene RPS13 mRNA content and normalizing to the mRNA content of the control group at D21 **(A)**, D28 **(B)** and D35 **(C)**, so all bars represent fold change compared to the control group. Bars (+SEM) without a common superscript differ significantly (*p* < 0.05). *n* = 8 (8 pens of 15 birds/pen).

There were significant (*p* < 0.05) treatment effects on the jejunal mRNA expression on days 21, 28, and 35 (Figure 3). On day 21, birds in the NE, FB + DON, and FB + DON + NE groups had lower claudin-1, claudin-2, and zona occludens-1 mRNA expression compared to the birds in the control group. On days 28 and 35, birds in the NE group had similar claudin-1 and zona occludens-1 mRNA expression when compared with the control group, but birds in the FB + DON and FB + DON + NE groups still had downregulated claudin-1 and zona occludens-1 mRNA compared to the control group. Similar trends were observed on days 28 and 35.

### Effect of Subclinical Dose of FB + DON and *E. maxima/C. perfringens* Challenge on Cytokine mRNA Expression

**FIGURE 4:**
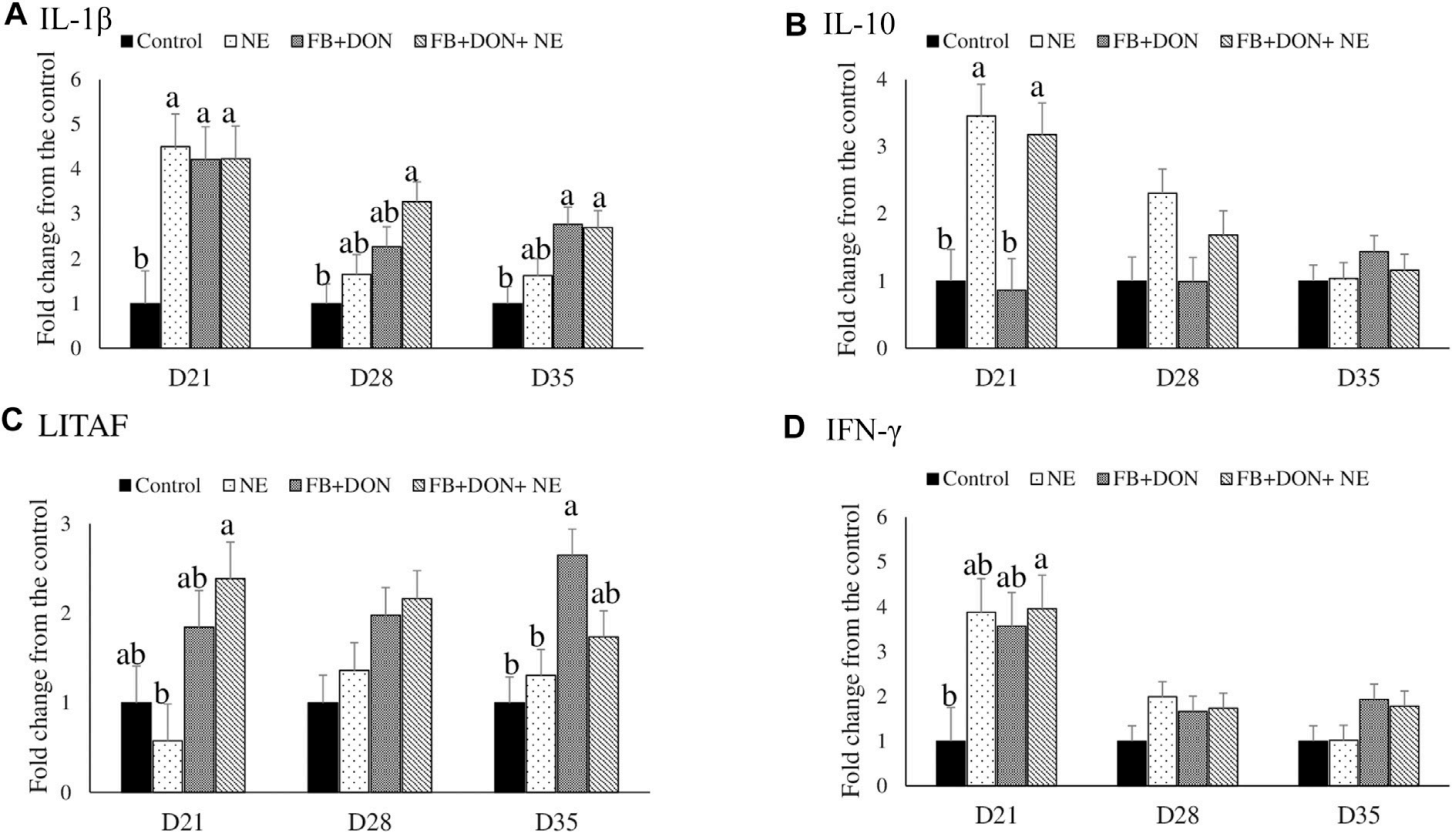
Effect of subclinical dose of FB + DON and *E. maxima/C. perfringens* challenge on cecal tonsil cytokine mRNA expression. Day-old chicks were distributed into four treatment groups: control, necrotic enteritis (NE), fumonisin + deoxynivalenol (FB + DON), and FB + DON + NE groups. Birds in the NE and FB + DON + NE groups received 2.5 × 10^3^ *Eimeria maxima* oocyst per bird on day 14. All birds received 1 × 10^8^ CFU/bird of *Clostridium perfringens* on days 19, 20, and 21. IL-1β, IL-10, LITAF, and IFN-γ mRNA content was analyzed after correcting for the housekeeping gene RPS13 mRNA content and normalizing to the mRNA content of the control group, so all bars represent fold change compared to the control group. Bars (+SEM) without a common superscript differ significantly (*p* < 0.05). *n* = 8 (8 pens of 15 birds/pen).

There were significant (*p* < 0.05) treatment effects on the cecal tonsil IL-1β, IL-10, LITAF, and IFN-γ jejunal mRNA expression on day 21 (Figure 4). On day 21, birds in the NE, FB + DON, and FB + DON + NE groups had an approximately 4-fold increase in IL-1β mRNA compared to the birds in the control group. Similar trends were observed on days 28 and 35.

On day 21, birds in the NE and FB + DON + NE groups had an approximately 3-fold increase in IL-10 mRNA compared to the birds in the control group.

On day 21, birds in the FB + DON + NE group had higher LITAF mRNA compared to the birds in the NE group. On day 35, birds in the FB + DON group had higher LITAF mRNA compared to the birds in the control group.

On day 21, birds in the FB + DON + NE group had an approximately 4-fold increase in IFN-γ mRNA compared to the birds in the control group.

### Effect of Subclinical Dose of FB + DON and *E. maxima/C. perfringens* Challenge on the Spleen and Cecal Tonsil CD8^+^: CD4^+^ Ratio

**FIGURE 5:**
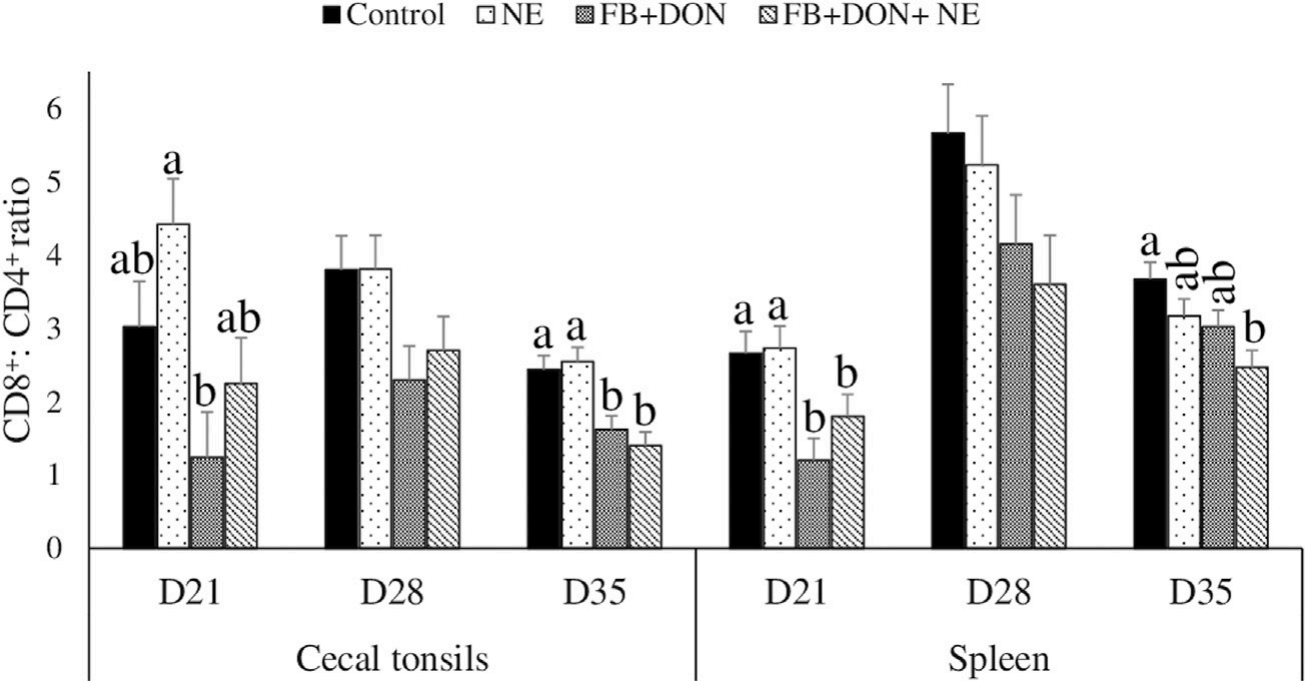
Effect of subclinical dose of FB + DON and *E. maxima/C. perfringens* challenge on the spleen and cecal tonsil CD8^+^: CD4^+^ ratio. Day-old chicks were distributed into four treatment groups: control, necrotic enteritis (NE), fumonisin + deoxynivalenol (FB + DON), and FB + DON + NE groups. Birds in the NE and FB + DON + NE groups received 2.5 × 10^3^ *Eimeria maxima* oocyst per bird on day 14. All birds received 1 × 10^8^ CFU/bird of *Clostridium perfringens* on days 19, 20, and 21. CD4^+^ and CD8^+^ cells were identified using fluorescent-linked anti-chicken CD4 and CD8 in a flow cytometer. Bars (+SEM) without a common superscript differ significantly (*p* < 0.05). *n* = 8 (8 pens of 15 birds/pen).

On day 21, birds in the FB + DON group had a lower CD8^+^: CD4^+^ ratio in the cecal tonsils compared to the birds in the control group (Figure 5). On day 35, birds in the FB + DON and FB + DON + NE groups had a lower CD8^+^: CD4^+^ ratio in the cecal tonsils compared to the birds in the control group

On day 21, birds in the FB + DON and FB + DON + NE groups had a lower CD8^+^:CD4^+^ ratio in the spleen compared to that in the birds in the control group. On day 35, birds in the FB + DON + NE group had a lower CD8^+^:CD4^+^ ratio in the spleen compared to the birds in the control group.

### Effect of Subclinical Dose of FB + DON and *E. maxima/C. perfringens* Challenge on Jejunal and Ileal Histomorphology

**FIGURE 6:**
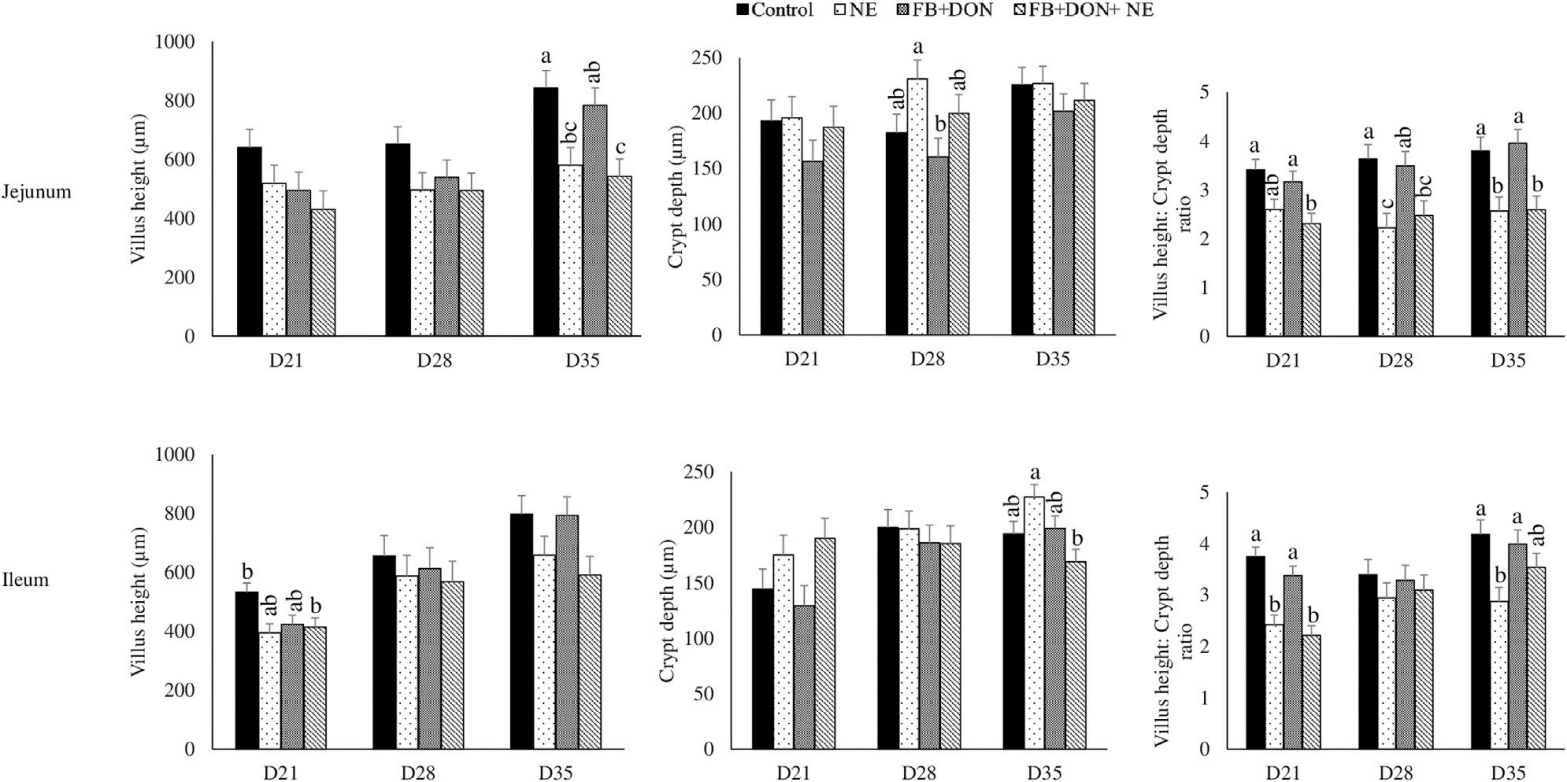
Effect of subclinical dose of FB + DON and *E. maxima/C. perfringens* challenge on jejunal and ileal histomorphology. Day-old chicks were distributed into four treatment groups: control, necrotic enteritis (NE), fumonisin + deoxynivalenol (FB + DON), and FB + DON + NE groups. Birds in the NE and FB + DON + NE groups received 2.5 × 10^3^ *Eimeria maxima* oocyst per bird on day 14. All birds received 1 × 10^8^ CFU/bird of *Clostridium perfringens* on days 19, 20, and 21. Jejunal and ileal sections were stained with hematoxylin and eosin. Villi height and crypt depth were measured using cellSens Imaging software, and villi height:crypt depth ratio was calculated. Bars (+SEM) without a common superscript differ significantly (*p* < 0.05). *n* = 8 (8 pens of 15 birds/pen).

On day 21, birds in the NE group had a 24% decrease (*p* > 0.05) in villi height to crypt depth ratio compared to the birds in the control group and the presence of FB + DON in NE-induced birds further decreased the villi height to crypt depth ratio by 8.4% when compared with NE group (Figure 6). Similar results were observed in the ileum on day 21.

### Effect of Subclinical Dose of FB + DON and *E. maxima/C. perfringens* Challenge on *C. perfringens*, *Lactobacillus* spp.*,* and *Bifidobacterium* spp. Loads in the Cecal Content

**FIGURE 7:**
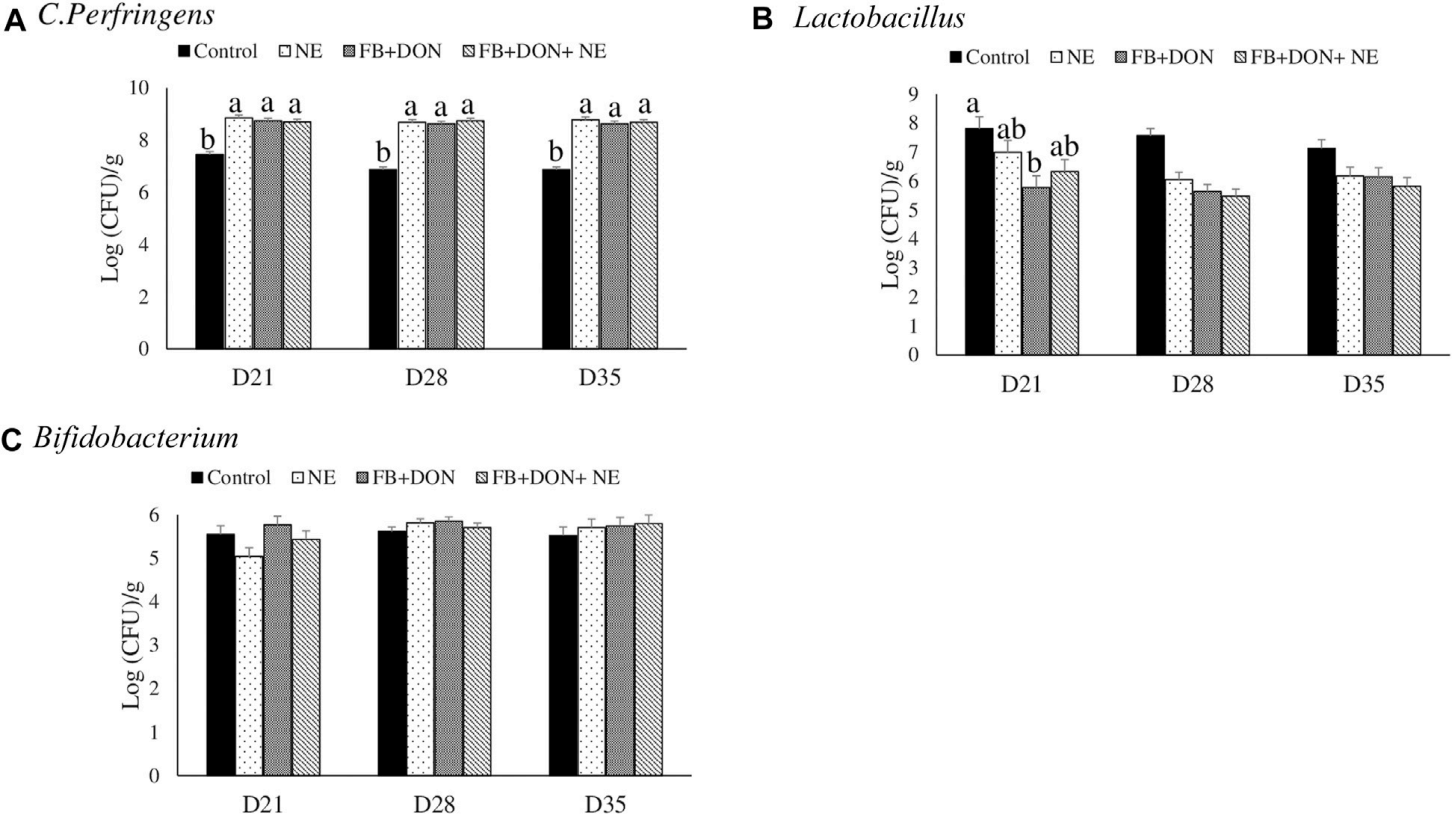
Effect of subclinical dose of FB + DON and *E. maxima/C. perfringens* challenge on *C. perfringens*, total *Lactobacillus,* and total *Bifidobacteria* loads in the cecal content. Day-old chicks were distributed into four treatment groups: control, necrotic enteritis (NE), fumonisin + deoxynivalenol (FB + DON), and FB + DON + NE groups. Birds in the NE and FB + DON + NE groups received 2.5 × 10^3^ *Eimeria maxima* oocyst per bird on day 14. All birds received 1 × 10^8^ CFU/bird of *Clostridium perfringens* on days 19, 20, and 21. Cecal content was analyzed for *C. perfringens*, **(A)** total *Lactobacillus,* **(B)** and total *Bifidobacteria* **(C)** through PCR. Bars (+SEM) without a common superscript differ significantly (*p* < 0.05). *n* = 8 (8 pens of 15 birds/pen).

On days 21, 28, and 35, birds in the NE, FB + DON, and FB + DON + NE groups had an approximately 1.3 Log increase in *C. perfringens* loads in the cecal tonsils compared to the birds in the control group (Figure 7).

On day 21, birds in the FB + DON group had lower (*p* < 0.05) *Lactobacillus* spp. compared to the birds in the control group.

### Heat Map Representing Pearson’s Correlation Coefficient Matrix Between Cytokine Amounts and Body Weight Gain

**FIGURE 8:**
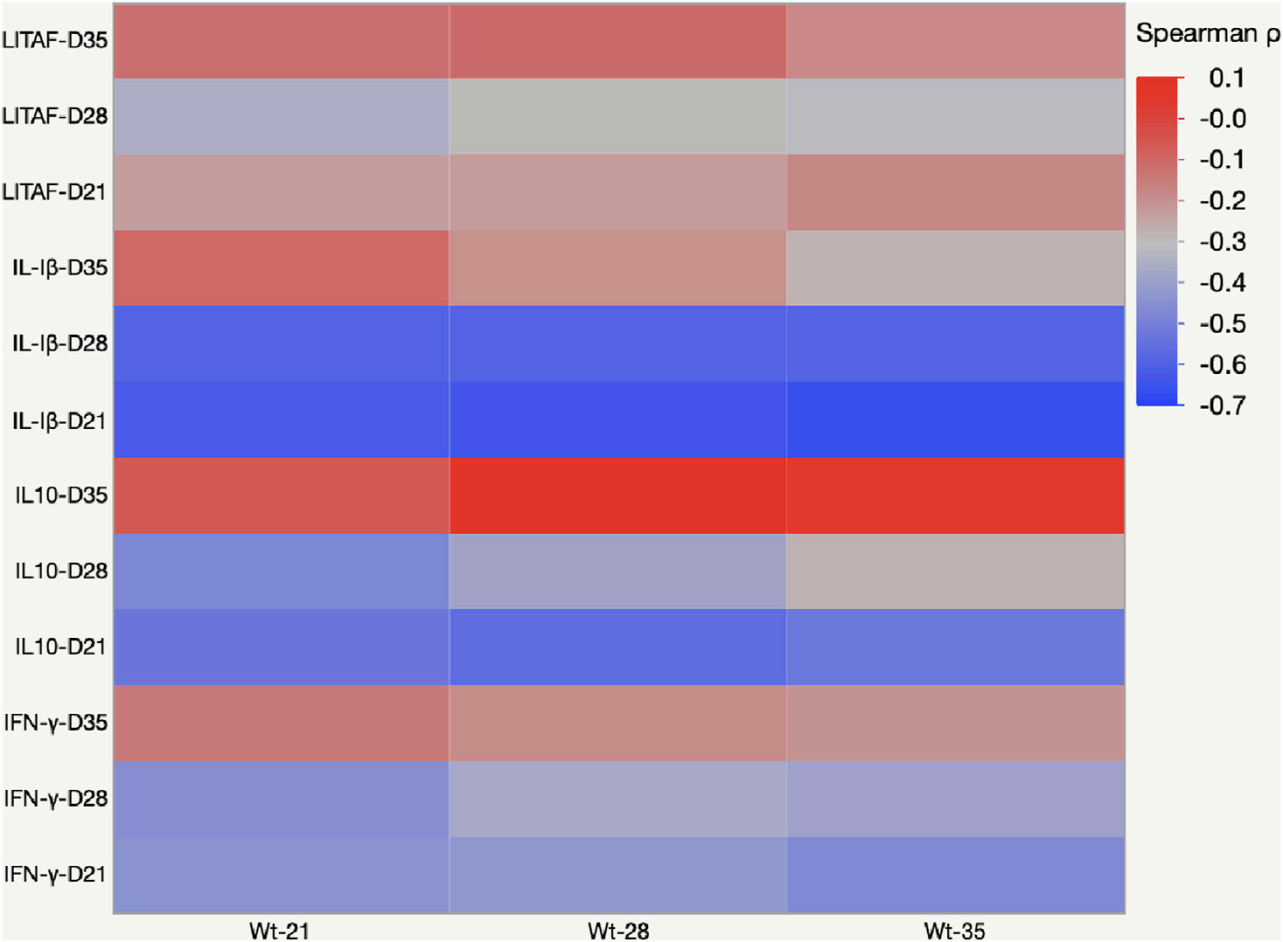
Heat map representing Pearson’s r correlation coefficient matrix between cytokine amounts and body weight gain. Heat map showing the transcriptional fold change of LITAF, IL-1β, IL-10, and IFN-γ in the cecal tonsils of birds fed mycotoxin contaminated diet and induced with necrotic enteritis. The color scale, −1 (blue) to +1 (red), and the negative value of the coefficient indicate that increased IL-1β and IL-10 mRNA expression levels are inversely affected the body weight.

The negative value of Pearson’s coefficient indicated that IL-1β and IL10- mRNA expression on days 21 and 28 were inversely related to body weight (Figure 8).

## Discussion

Corn is the major energy source in poultry feed and constitutes 50%–80% of the finished poultry feeds in the United States and Europe (Guerre, 2016). Mycotoxins are ubiquitous in nature (Shimshoni et al., 2013), and under practical conditions, it is difficult to produce clean corn without mycotoxin contamination. In this study, the starter basal diets in the control group were naturally contaminated with 40 μg/kg aflatoxin, 400 μg/kg FB, and 100 μg/kg DON, and the finisher basal diets in the control group were contaminated with 3 μg/kg aflatoxin, 1,500 μg/kg FB, and 200 μg/kg DON. Because there is an increase in the occurrence of mycotoxins contamination of poultry feed under field conditions, there is a growing concern regarding the negative effects of combined mycotoxins, even when present at sub-clinical doses, on gut health. Hence, this study aimed to identify whether the combined presence of FB and DON at subclinical concentration predisposed broiler chickens to NE and acerbated the severity of NE lesions.

In the current study, a combined dose of 3 mg/kg FB and 4 mg/kg DON decreased the chickens’ body weight on day 14 even before the birds were inoculated with *E. maxima.* In birds that were induced with NE, FB and DON further decreased the body weight gain. Our data suggest that a combined dose of 3 mg/kg FB and 4 mg/kg DON in the poultry diet increased gut permeability and decreased villi height to crypt depth ratio, which can be expected to decrease body weight and increase the FCR. An earlier study identified that combination of FB and DON either at 20 and 1.5 mg/kg or 20 and 5.0 mg/kg feed, respectively, increases the feed conversion ratio. A similar result was observed in piglets when feeding 6 mg/kg FB and 3 mg/kg DON in combination, which decreased the production performance (Grenier and Oswald, 2011). Broilers exposed to multiple mycotoxins at subclinical doses in the starter to finisher diets exhibit decreased production broiler performance and impaired health (Wang et al., 2005). Earlier reports have identified that poultry feed contaminated with 5 mg/kg DON alone did not alter the chicken production performance (Awad et al., 2011). Similarly, FB alone at 300 mg (Brown et al., 1992) or 50 mg (Yu et al., 2022) did not cause a decrease in production performance in broiler birds. Considering that when FB or DON was individually fed, they did not decrease the production performance even when present at 300 mg/kg and 5 mg/kg. It should be noted that subclinical doses of FB + DON had numerical changes, rather than statistical significance, on production performances. It has been suggested that the interpretation of *p* values should not be a dichotomous conclusion as either significant or nonsignificant, but it should be interpreted based on the real-world implication of the observed change in the data points (Andrade, 2019). FB + DON decreased the body weight gain by 87 g, further decreased the body weight by 97 g, and worsened the FCR by 3 points in birds induced with NE on day 35. FB + DON at subclinical dose can thus lead to a loss of up to 184 g per bird, which accounts for approximately 10.5% of live body weight. Thus, it can be concluded that in this present study, the combined dose of DON and FB had a synergistic negative effect on body weight gain and feed conversion ratio.

The presence of both FB and DON increased the severity of the NE lesions in birds induced with NE. However, FB and DON did not increase NE mortality (23.3% and 21.2% mortality in FB + DON and FB + DON + NE group). In the NE model studied, the control group was inoculated with *C. perfringens* and, hence, the *C. perfringens* loads were approximately 7 logs/g of cecal content. In the absence of accompanying intestinal wall damage because of *E. maxima* or mycotoxins, the control group had no NE lesions. These findings suggest that combined subclinical doses of FB and DON increase the severity of the NE lesion without increasing the associated mortality. NE lesion scores, but not the associated mortality, should be used to assess the cost of subclinical doses of FB and DON under field conditions.

Previous studies have identified that chronic exposure to FB1 at 100 mg/kg concentration for 28 days or 300 mg/kg for 14 days decreases the jejunum villus height and villus: crypt depth ratio and causes mild villus atrophy and goblet cell hyperplasia in broiler chicks (Rauber et al., 2013). This study identified that the presence of FB and DON combination decreased villi height to crypt depth ratio similar to that in the NE group. The villi length to crypt depth ratio is an indicator of the intestinal renovation rate and a higher villi to crypt ratio indicates a lower intestinal turnover (Brown et al., 1992; Van Nevel et al., 2005). Thus, subclinical doses of FB and DON combination increased the intestinal turnover and contributed to the observed decrease in FCR and loss in body weight gain during NE.

FB and DON acerbated the loss in the tight junction protein and increase in gut permeability associated with NE. FB and DON combination decreased the jejunal claudin-1, claudin-2, and zona-occluden-1 in the intestine. The decrease in the jejunal tight junction protein owing to subclinical mycotoxin was comparable to the loss in the tight junction in the birds induced with NE. Earlier studies have identified that chronic exposure to FB decreases the proliferation of intestinal epithelial cells and breaks down the gut barrier in pigs (Bouhet et al., 2004). Tight junction proteins are comprised of transmembrane proteins such as claudins and occludens, and cytoplasmic proteins, such as zona occludens (Findley and Koval, 2009). Tight junction proteins act as a barrier to pathogens and harmful toxins while permitting the entry of nutrients, ions, and water (Tomaszewska et al., 2021). Caco-2 cells exposed to a combination of aflatoxin and ochratoxin had significantly decreased tight junction proteins (Gao et al., 2018). FB inhibits ceramide synthase, which results in the accumulation of sphingoid bases and their metabolites, leading to the depletion of complex sphingolipids (Wang et al., 1991). In addition, FB leads to the accumulation of sphinganine (Riley et al., 1999) and increases calmodulin, an apoptotic protein. Alteration in the sphingolipid metabolic products, sphingosine content, and calmodulin can be expected to decrease intestinal cell viability and loss in tight junction proteins (Bouhet et al., 2004). Furthermore, chronic exposure to FB + DON enhances the claudin-1, claudin-2, and zona-occluden internalization by endocytosis (Fujita et al., 2000). This results in the reduction of claudins at a cellular level and a lack of new molecules to replace the damaged tight junction proteins (Hopkins et al., 2003).

During NE infection, the integrity of intestinal epithelial cells is compromised due to either inflammation or toxins or the associated gut dysbiosis. Quantification of serum FITC-d is commonly used as an indicator for assessing intestinal paracellular permeability and magnitude of severity (Kuttappan et al., 2015). The oral administration of FITC-D passes through the disrupted intestinal epithelium and enters systemic circulation, which can be quantified in the blood (Liu et al., 2021). In this current study, the presence of FB and DON caused a loss in gut integrity, and this loss in gut integrity was acerbated in birds challenged with NE. The observed increase in serum FITC-D level correlated with decreased tight junction proteins in the ileum. A decrease in the tight junction proteins of the intestine leads to a loss in gut integrity and an increase in gut permeability, and it can explain the observed increase in serum FITC-D concentration. This current study suggests that chronic exposure to even subclinical doses of mycotoxins could adversely damage the intestinal gut epithelium.

FB and DON increased the cecal tonsil IL-1β, an inflammatory cytokine. Upregulation of interleukins is observed normally during various bacterial and parasitic infections (Mensikova et al., 2013). Immune system activation includes changes in cytokines such as tumor necrosis factor (TNF-α, IL-1β, IFN-γ, and IL-10 (Wallach et al., 2014). Activated macrophages secrete IL-1β to induce inflammation (Bhat and Fitzgerald, 2014). In mice, a single dose of *in vivo* DON exposure increases TNF-α, IL-1β, IFN-γ, and IL-10 in CD4^+^ cells isolated from spleen and Peyer’s patches (Zhou et al., 1997). *In vitro* treatment of chicken splenocytes with DON increases the concentrations of IL-1β, IL-10, and IFN-γ (Azcona-Olivera et al., 1995; Ren et al., 2015). In this present study, birds exposed to FB and DON had increased cecal tonsil IFN-γ mRNA transcription at levels similar to that in the birds undergoing a NE challenge. IFN-γ plays an important role in the host’s defense against intracellular pathogens such as coccidiosis. This increased IFN-γ mRNA transcription at D21 in the combined toxin group suggests that FB and DON could have had a synergistic effect on IFN-γ mRNA transcription. Cecal tonsils of *Eimeria-*challenged birds had an increase in IFN-γ mRNA transcription when chickens were fed *Fusarium* mycotoxins contaminated diet (Girgis et al., 2010). Chronic exposure to combined FB + DON activates the NF-kB pathway to upregulate pro-inflammatory cytokines (Pinton and Oswald, 2014; Taranu et al., 2015). Several studies have identified that the dietary mycotoxins, at doses even below EU guidance, could upregulate both pro and anti-inflammatory cytokines in the duodenum and jejunum (Bracarense et al., 2012; Lucke et al., 2018; Guo et al., 2021). Similarly, in this present study, 4 mg/kg DON and 3 mg/kg FB increased the pro-and anti-inflammatory cytokines, suggesting that combined toxins could have adverse effects on intestinal epithelial cells to modify the cecal tonsils cytokines expression in broilers. Furthermore, Pearson’s correlation analysis identified significant negative correlations (*p* < 0.05) between IL-1β, IL-10, and body weight. The negative coefficient indicated that the chronic exposure to mycotoxins increased the IL-1β and IL-10 mRNA transcripts, coinciding with an ultimate decrease in body weight gain. Activation of the immune system and cytokines production requires energy resources and affects the production performance, resulting in a trade-off between immune function and growth (van der Most et al., 2011). NE infection by itself increased proinflammatory cytokines, and further synergism between FB, DON, and NE acerbated the loss in body weight gain in the FB + DON + NE group.

T cell proliferation involves the activation and differentiation of T cells into effector and memory subsets which is critical for the adaptive immune system. CD8^+^: CD4^+^ ratio is a marker of immune dysfunction (Roitt, 1992; Martin et al., 2016). The impairment in CD4^+^ T cell regeneration and persistent elevation of CD8^+^ T cells are indicators of inflammation that involves gut microbial translocation (Hirakawa et al., 2020; Ruhnau et al., 2020). In this present study, the presence of FB + DON decreased the CD8^+^: CD4^+^ cell ratio in the cecal tonsils, and this effect was acerbated in the FB + DON + NE group compared to the control group. Similar results were observed in chickens’ peripheral mononuclear cells (PBMCs) when they were fed contaminated diets containing up to 3.8 μg/g deoxynivalenol (DON), 0.3 μg/g 15-acetyl DON, and 0.2 μg/g zearalenone (Girgis et al., 2008). Furthermore, broilers fed 20 mg/kg FB and 1.5 mg/kg of DON had an increased percentage of T lymphocytes, and CD4^+^CD25^+^ in the cecal tonsils (Grenier et al., 2016). In bovine and porcine PBMCs, a similar kind of trend was observed when they were fed DON contaminated diet. In porcine PBMCs, *in vitro* studies with DON at 0.4 mM or higher concentration have decreased the proliferation of CD8^+^ and CD4^+^ cells (Novak et al., 2018). Similarly, beef cattle exposed to 1.7 mg DON and 3.5 mg FB for 21 days has significantly decreased the CD8^+^: CD4^+^ ratio (Duringer et al., 2020; Roberts et al., 2021). Our studies demonstrated that combined subclinical doses of FB and DON negatively affected the proliferation of the CD8^+^ and CD4^+^ T cells. FB and DON target the cell with high protein turnover and inhibit protein synthesis. CD8^+^ and CD4^+^ cells are considered highly proliferative cells (Overgaard et al., 2015) and are likely highly sensitive to FB and DON (Taranu et al., 2010; Daenicke et al., 2011). Impaired CD8^+^ and CD4^+^ cell proliferation can be expected to compromise the immune response to NE. Changes in T-helper and cytotoxic T cell profiles, along with changes in inflammatory cytokines, suggest that the chicken immune system is altered by chronic exposure to *Fusarium* mycotoxins even at a subclinical dose in broiler chickens leading to impaired resistance to NE. Our results suggest that the CD8^+^: CD4^+^ ratio could be a potential biomarker of early *Fusarium* mycotoxin exposure.

*Lactobacillus* spp. and *Bifidobacterium* spp. are considered to be beneficial bacteria in the chicken gut. In this present study, the subclinical dose of FB and DON decreased the *Lactobacillus* spp. load in the ceca. Similar results were found when chickens were exposed to DON 5 mg/kg diet (Antonissen et al., 2015; Guo et al., 2021). Chronic exposure to subclinical doses of FB and DON increased the *C. perfringens* load and caused intestinal dysbiosis, and hence, this current study identified that FB and DON mycotoxins can be predisposing factors for *C. perfringens-*induced NE in chickens. Increased *C. perfringens* altered the balance between intestinal microbiota, with major changes observed in *Lactobacillus* spp. (Antonissen et al., 2016; Zhang et al., 2018; Hernandez-Patlan et al., 2019). The chronic exposure to FB + DON increased the cecal *C. perfringens* load but had no effect on *Bifidobacterium* spp. (Lucke et al., 2018). Therefore, it can be concluded that chronic exposure to subclinical doses of combined FB + DON affected the relative abundance of *Lactobacillus* spp. and exacerbated the NE by enhancing intestinal inflammation and shifting the gut microbiome towards pathogenic microorganisms (Yang et al., 2021).

The findings reported here have significant practical importance and reflect the real-world problem because of the common occurrence of *Fusarium* mycotoxins in poultry feeds and subclinical necrotic enteritis occurrence in the field. According to the FDA, the recommended level for FB and DON in the poultry finished diet is 50 mg/kg and 5 mg/kg (FDA, 2001; FDA, 2010). The level of FB and DON in the experimental diets of the current study was much lower than the FDA tolerance levels. The findings of this study represent the effects of chronic exposure to the subclinical levels of FB and DON in broiler chickens and their role in inducing subclinical necrotic enteritis. Our findings identified the mechanism through which FB and DON exhibited synergistic effects and predicted the specific thresholds of combined toxins and their adverse effects in chickens. Our data suggested that *Fusarium* mycotoxins not only directly affected the production performance but also influenced chicken health by inducing NE and acerbated the severity of NE.

Our data demonstrated that chronic feeding of a combined dose of 3 mg/kg FB and 4 mg/kg DON in the poultry diet downregulates the tight junction proteins and increased the severity of NE in broiler chickens. Chicken diets with FB and DON contamination, even at subclinical levels, induced a negative impact on performance, altered small intestinal morphology, and significantly increased the incidence of NE. In conclusion, the presence of FB and DON decreased the BWG, increased the FCR, increased gut permeability, decreased jejunal tight junction protein, increased inflammatory cytokines in the cecal tonsil, decreased CD8^+^:CD4^+^ ratio in the cecal tonsil and spleen, increased *C. perfringens* load in the cecal content, and decreased *Lactobacillus* spp. loads in the cecal content and predisposed broiler birds to NE.

## Data Availability

The original contributions presented in the study are included in the article/Supplementary Material; further inquiries can be directed to the corresponding author.
